# Awareness on good and bad touch among school children in north Gujarat, India

**DOI:** 10.6026/97320630019849

**Published:** 2023-08-31

**Authors:** Mahalakshmi Beeman, Ramalakshmi Govindan, Kiritkumar Patel Tasvirben, Nagooran Siva Subramanian, Zakirhusen MahidaSadiya, Kirti Harshal

**Affiliations:** Nootan College of Nursing, Sankalchand Patel University, Visnagar, Gujarat -384315, India; College of Nursing, S.G.R.R University, Dehradun, Uttarkhand - 248001, India

**Keywords:** Awareness, good touch, bad touch, school children.

## Abstract

It is not common to discuss child sexual assault in open conversation. Hence, it is vital to explain the distinction between good and harmful contact in order to stop harmful touch and preserve the safety of children. Therefore, it is of interest to assess the efficacy of teaching initiatives addressing appropriate and inappropriate touching with a view to reducing child sexual abuse. Thus, an experimental research design was employed. Further, a single group pre-test and post-test design was chosen. The data collected from 60 school children at Visnagar, Gujarat, India. Data shows that the mean post-test knowledge score, which averaged at 16.03, was higher than the mean pre-test knowledge score (5.6). Thus, educational programs on child sexual abuse can significantly improve awareness among school children.

## Background:

In India, child sexual abuse (CSA) is a significant and little recognized issue. Despite being such a serious issue, there is a culture of silence surrounding CSA. The only way to solve the hidden issue of CSA is to provide kids with the tools they need to protect them and report abuse [1].The problem is that they even do not realize that something wrong had happened to them as they do not know about good touch and bad touch. In India child sexual abuse is widely spreading and occurs at both within and outside family circle. These negative consequences of touch effect children's mental health throughout their lifespan [2]. The ability to distinguish between a good and terrible touch is essential and can be quite valuable in today's society. A good touch makes one feel comfortable and well-treated. A bad touch, on the other hand, is essentially one that makes you feel apprehensive, uneasy, or afraid. Since a terrible touch can also end in child sexual abuse, it is crucial that as many people as possible are aware of this issue. There are many bad people in the world who like exploiting children's naivety [3].The study shows the results that there was a significant difference in adolescent girl's understanding of sexual exploitation after participating in a video-assisted teaching program (t test, 25.57, df=49, 0.05 level) [4]. The knowledge, attitude, and skills of children regarding preventing sexual abuse have been researched in the past in relation to child sexual abuse prevention initiatives. Despite the fact that many studies urge prevention program to use a variety of strategies, such as parent education and community awareness initiatives, little is known about how prevention program actually operate in their respective community contexts [5]. Children had a good memory for the commands and maintained them for a longer time. This was confirmed by a distinguished rise in knowledge scores [6]. Physical activity may have valuable benefits on cognitive development during early childhood, according to a comprehensive analysis of natural play activities [7]. Every community is affected by the multifaceted public health issue of sexual abuse. Primary-level preventive strategies for combating sexual abuse have a significant role to play. However, there is little evidence on parent involvement in child sexual abuse prevention strategies [8]. A study was conducted in Coimbatore revealed that found the majority of girls were ignorant of sexual abuse, its various forms, and what to do in the event of sexual assault. Therefore, it is crucial to develop an organized education program to teach kids about child sex abuse in order to stop it from happening. Children can avoid these types of tragedies by learning about body safety guidelines and being given the authority to report incidents [1]. Therefore it is of interest to investigate the efficacy of education program among school children regarding "good touch" and "bad touch".

## Methodology:

The current study's objective was to assess the efficacy of teaching initiatives addressing good and bad touch in order to decrease child sexual abuse. For this study, an experimental research design was employed. For this investigation, a single group pretest and posttest design was chosen. The information was gathered from 60 school-age youngsters in a few of Visnagar, Gujarat's schools. Purposive sampling was used to choose the samples. Through videos, the kids were taught about good touch and bad touch. The teaching program included various aspects like identifying good touch and bad touch, how to react to a bad touch, whom to inform about bad touch etc. A pre- and post-test structured questionnaire is used. After a seven-day teaching program, a post-test was administered. The mean, standard deviation, and chi square test were used in the descriptive and inferential statistics used to examine the data.

## Results:

In demographic characteristics the data shows that 14(23.3%) of were in the age of 7-8 years, 25(41.6%) of school children were in the age of 9-10 years, 21(35%) of school children were in the age 11-12 years. Majority 36(60%) of them were female and 24(40%) of them were male. Twenty (33.33%)of school children from primary, 10(16.66%) of school children from secondary education, 25(41.66%) of school children from high secondary mother education status. 14(23.33%) of school children from 3rd-5th class of study and 20(33.33%) of school children from 6th -7th class of study, 26(43.33%) of school children of 8th -9th class of study 28 (46.66%) of schoolchildren are parents of one child, 9 (15%) are parents of two children, 15 (25%) are parents of three children, and 8 (13.33%) are parents of many children. 23(38.33%) of school children belongs to nuclear family and 37(61.66%) school children to joint family. 27(45%) of school children living in rural area and 23(55%) of school children living in urban area. Knowledge score shows that 42 (70%) School children score between 0-7 which shows inadequate knowledge toward good touch and bad touch. 18(30%) school children score between 8-14 which shows moderate knowledge toward good touch bad touch, none of school children score between 15-20 which shows no one having adequate knowledge toward good touch and bad touch. Here, it can be interpreted that highest percentage 70% of school children had inadequate knowledge regarding good touch and bad touch. Data analysis revealed that the mean pre-test score was 5.6 and the mean post-test score was 16.03. The mean difference was 10.43. The pre-test knowledge score's standard deviation was 3.33, whereas the post-test knowledge score's SD was 2.07. The estimated t value was 20.61, the significant level was NS, the DF value was 59, and the p value was 1.671. Here, it can be inferred that the estimated 't' value is larger than the tabulated 't' value, demonstrating the efficacy of video-assisted instruction in enhancing students' knowledge of good touch and bad touch. The correlation between demographic factors and knowledge score reveals a strong relationship between age, gender, religion, and mother status, class of study, monthly income and number of children, family structure, and place of living.

**Figure 1:**
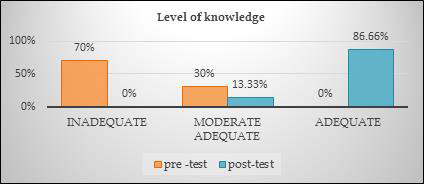
Bar shapes diagram showing percentage distribution of the sample according to the pre-test and post -test level of knowledge.

Figure 1: Shows that 70% of the sample's had insufficient knowledge before the video education programme was administered. In the post-test, there was a noticeable improvement in the sample's knowledge; 13.33% of the school children gaining moderate adequate knowledge and (86.66%) gaining adequate knowledge that were above average. The significant difference between schoolchildren's pre-test and post-test knowledge scores about good touch and bad touch can be shown here, so accept hypothesis 1 (H1). The data presented in Table 1 shows that the mean knowledge score after the exam (16.03) was greater than the mean knowledge score before the test (5.6). The estimated value (20.61) was higher than the value (1.67) at the 0.5 level of significance, demonstrating that the video-assisted training was successful in enhancing student's awareness of appropriate and inappropriate touch.

## Discussion:

The present study found that there is significant difference between pre-test knowledge score and post-test knowledge score of school children regarding good touch and bad touch. Hence the educational programme was found to be significant in increasing awareness among school children. This study result is supported by another study conducted in Coimbatore, Tamil Nadu. The study was designed to evaluate the effectiveness of health education on awareness of child sex abuse among school children following the intervention, there was a considerable increase in understanding about the laws protecting children from sexual offences, going from 49% to 78% [1]. In order to understand the impact of school-based child abuse prevention program on awareness about child abuse, a meta-analytic review was conducted. The inclusion of k = 37 papers published between 1985 and 2019 brought the total to 37, with k = 34 studies reporting on the impact of school-based interventions on knowledge of child abuse [9].

The 5-week preventative program has five modules, including ones on emotional awareness, good contact versus harmful touch, and body safety guidelines. Using a latent Markov analysis, the efficacy of the preventative program was looked into. The ideal model was determined to be a three-class solution, with Status-1 (the self-protecting group), Status-2 (the risky secret keepers), and Status-3 (the risk group). A two-month follow-up analysis revealed that the knowledge and skill gains were kept up. This program should be taken into consideration as a feasible strategy for addressing the demand for preventive measures against child sexual abuse in Turkish preschool curricula [10].

To gather information regarding the outcomes of program designed to prevent child sexual abuse, another study was carried out. A meta-analytic method was used to calculate the post-test and follow-up effect sizes of 16 evaluation studies of school program intended to reduce the incidence of child sexual abuse victimization. There were found to be significant and significant mean post-intervention (d =.71) and follow-up (d =.62) effect sizes, suggesting that victimization prevention program are successful at teaching youngsters about sexual assault and how to defend themselves [11]. Another randomized-control trial for children aged 8-10 years,to evaluate the impact of educational games regarding prevention of child sexual abuse. Children in the experimental group, significantly (p < .001) increased their knowledge scores; whereas those in the control group did not [12]. Intervention programs for child sexual abuse at schools were created to teach students how to keep themselves safe from sexual abuse. 29 randomized control trial and quasi-experimental studies from various nations were examined in total. Standardized mean difference (SMD) and 95% confidence intervals (CI) of -1.06 (95%CI: -1.29, -0.84), -0.91 (95% CI: -1.2, -0.61), and -0.51 (95% CI: -3.61, 0.58), respectively, were used to compare knowledge, abilities, and attitude between pre- and post-intervention groups. The SMD of knowledge was 0.9 (95% CI: 0.63, 1.18), of skills was 0.39 (95% CI: 0.07, 0.71), and of attitude was 1.76 (95% CI: 0.46, 3.07) for comparisons between the intervention and control groups. The programs were found to be successful at enhancing the students' knowledge, abilities, and attitudes from before the intervention to after it, as well as between the intervention and control groups [13]. There are now many different types of initiatives to prevent child sexual abuse. The difference between a good touch and a bad touch should be spread. This is a challenging topic for young children to learn and maintain, evaluation study has shown [14].

The percentage of kids (94.5%) who agreed they would report bad touching to a trusted adult until that person believed them showed a considerable improvement. Only 31% of kids knew what to do if they were being sexually abused prior to the intervention; after the intervention, this number considerably increased. Following the intervention, there was a considerable increase in understanding about the laws protecting children from sexual offences, going from 49% to 78%. Nearly 90% of kids said they were aware of the child help line's phone number [15]. The experimental group was subjected to the "Don't Touch My Body" Training Program (DTMB-TP), which was delivered in person over the course of one class hour (40 minutes). In the intervention group, knowledge of the kids' private body parts, good/bad touch, reactions to strangers, and the family security password increased statistically significantly (p .05) in the post-test. In the post-test, there was no improvement in the CSA knowledge level score for the control group (p >.05). The DTMB-TP successfully raised participants' awareness of sexual abuse prevention. This study came to the conclusion that education successfully increased awareness to prevent CSA and supplied the foundational information for more extensive studies [16].

Sixty-two percent of the sample said they had taken part in a school-based "good touch-bad touch" program to avoid sexual assault. In comparison to 14% of respondents who never reported having a preventative program, 8% of respondents who said they had ever participated in one said they had later been sexually molested. In terms of behavioural measures of sexual activity, there were no changes between respondents who had and hadn't taken part in a preventative program [17].

## Conclusion:

In this scenario, child sexual abuse (CSA) is a significant and delicate issue. The children are not much aware about good and bad touch in surrounding and common areas. Moreover, they are not reporting the incident to their parents or teachers. With a view to importing knowledge and preventing the incident of child sexual abuse, the current study was conducted and the Data analysis revealed that the mean post-test score was (16.03) higher than the mean pre-test score (5.6) where the mean difference was 10.43. The t value was 20.61and it proved that video-assisted teaching is effective. This shows that the kids learned enough about good and bad touching.

## Figures and Tables

**Table 1:** Mean, S.D, Mean difference and 't' value of pre-test and post-test knowledge score of effectiveness of video assisted teaching knowledge. DF = n-1(60-1) =59

**Parameter**	**Mean**	**Standard deviation**	**Mean difference**	**'t'value**	**Table 't' value**	**Level of Significance 0.05**
Pre-test	5.6	3.33	10.43	20.61	1.67	S
Post-test	16.03	2.07				
